# A Study to Analyze Different Patterns of Quid Usage among Subjects with Oral Submucous Fibrosis in Mangalore Population

**DOI:** 10.1155/2016/6124059

**Published:** 2016-09-29

**Authors:** Vidya A. Holla, L. K. Chatra, Prashanth Shenai, Devika Shetty, Ashwini Baliga

**Affiliations:** ^1^A J Institute of Dental Sciences, Mangalore, India; ^2^Department of Oral Medicine and Radiology, Yenepoya Dental College, Mangalore, India

## Abstract

*Aim and Objectives*. Oral submucous fibrosis (OSF) is a potentially malignant disorder associated with the usage of areca nut. Usage of processed forms of areca nut is popular among the youth and its carcinogenic effects are not well known. Due to large immigrant population, various patterns of areca nut usage are seen. The aim of this study is to assess the various quid chewing patterns and their association with severity of OSF.* Materials and Methods*. A cross-sectional study was carried out with 250 cases clinically and histologically diagnosed as having OSF lesion that were selected and subjected to a detailed habit history which was recorded through preformed questionnaire. The data obtained was statistically analyzed.* Results*. Among the 250 subjects, males were seen to be affected more than females within the age group of 26–35 years and were having clinical stage I OSF. A combination of processed areca nut and processed tobacco was used by the majority of the subjects with duration of 1 to 5 years, at a frequency of 3 to 5 quids per day.* Conclusion*. The present study confirms the association between oral submucous fibrosis and the quid containing processed areca nut and processed tobacco and also highlights the increasing youth population using the processed forms of areca nut.

## 1. Introduction

Habits associated with the oral cavity can have deleterious effects on the oral health and affect the normal functioning of the oral cavity. Some habits are developed as a leisure activity and some are imbibed as a cultural practice. Either way, they become addictive and can have far reaching effects on oral as well as general health.

Chewing areca nut has gained popularity due to social acceptability, religious beliefs, perceived health benefits, and addiction [1]. Regional variations in the type of areca nut preparations used and the varied patterns of chewing using them have led a specific site in the oral cavity to be involved [2]. The areca nut is either chewed alone or along with betel leaf and other ingredients such as lime, catechu, spices, or sweeteners and used either as traditional pan or commercially used gutkha [3].

Usage of areca nut has been the single-most important etiological factor considered for development of OSF [4]. The disease is predominantly seen in India, Bangladesh, Sri Lanka, Pakistan, Taiwan, China, and among other Asians, with a reported prevalence ranging up to 0.4% in Indian rural population [2].

Mangalore, which is located at the border of Karnataka and Kerala, is a predominant areca nut growing region and has people from both states residing in it as well as a large number of immigrant population. Therefore, an attempt is made in this study to assess the various chewing patterns associated with the severity of oral submucous fibrosis (OSF).

## 2. Material and Methods

The present cross-sectional study was carried out wherein 250 cases of OSF were selected among the patients attending our institution and those attending the rural dental camps as per the clinical criteria and staging set by Haider et al. [5] (Table 1).

An incisional biopsy was done for histopathological confirmation after an informed consent was obtained. The patients were later subjected to a questionnaire regarding the various patterns of chewing habit and the quid constituents used. The data obtained from the procedures were tabulated and statistically analyzed.

## 3. Results

**Figure 1:**
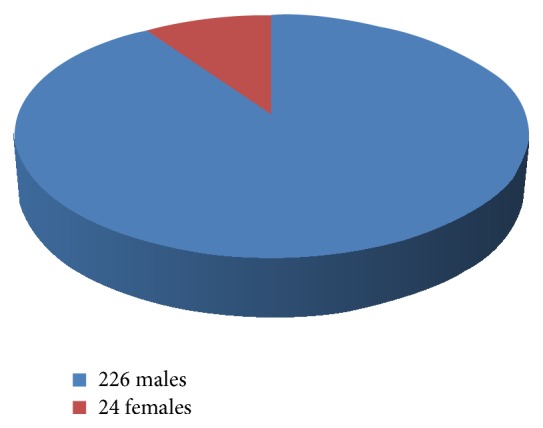
Gender distribution.

**Figure 2 fig2:**
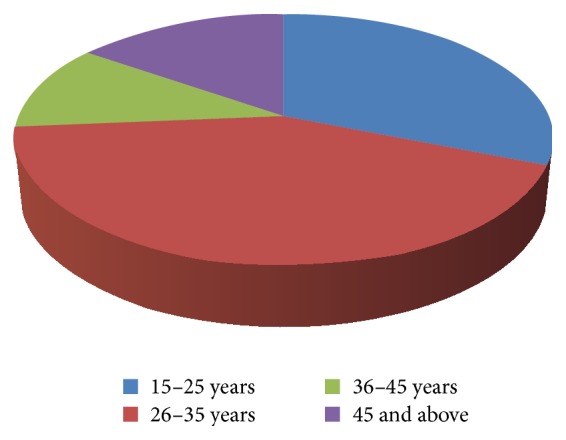
Age distribution.

**Figure 3:**
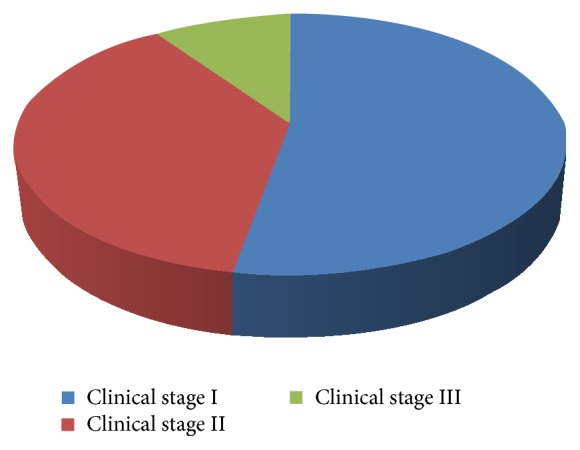
Cases distributed based on clinical staging.

The data collected was analyzed and the results were obtained and the following observations were made and summarized in Figures 1 [Fig fig2]–3 and Tables 1 [Table tab2] [Table tab3] [Table tab4] [Table tab5] [Table tab6] [Table tab7] [Table tab8]–9.

Out of the 250 OSF patients examined, 90.4% were males (Figure 1) and 31.2% of the cases were in the age of 15–25 years and 42.4% of the cases were in the age group of 26–35 years (Figure 2). Among the 250 cases, 52.8% of the cases were of clinical stage I, 37.6% of the cases were of clinical stage II, and 9.6% of the cases were of clinical stage III (Figure 3).

Among the 250 cases included in the study, 5.6% of the cases chewed the type II quid, 7.2% of the cases chewed type III quid, 30.4% of the cases chewed type IV quid, 39.2% of the cases chewed type V quid, and 17.6% of the cases had multiple quid chewing habits (Table 2).

Among the 14 cases who chewed type II quid, 85.7% of the cases were having OSF stage I and 14.2% of the cases were having OSF stage II. Among the cases who were chewing type III quid, 66.6% were having OSF stage I and 33.3% of the cases were having OSF stage II. Out of the 76 patients using type IV quid, 57.8% of the cases were having OSF stage I, 31.5% of the cases were having OSF stage II, and 10.5% of the cases were having OSF stage III. Among the 98 cases that were using type V quid, 42.8% of the cases were having clinical stage I OSF, 42.8% of the cases were having clinical stage II OSF, and 14.2% of the cases were having clinical stage III OSF. In this present study maximum patients were having clinical stage I OSF and used type V quid and this was found to be statistically significant (Table 3).

Among the cases seen in clinical stage I, the majority of the cases chewed 3–5 times daily and were found to use type IV quid rather than the traditional quid forms. This was found to be statistically significant (Table 4).

In clinical stage I, the majority of the cases were using quid for duration of 1–5 years, and the processed forms of quid are seen to be the cause. This association between the type of quid and duration was found to be very highly significant (Table 5).

In clinical stage II, the majority of the cases were using the quid 3–5 times daily, and the cases using the processed forms have manifested with OSF rather than with the use of the traditional type III quid. This association between the type of quid and duration was found to be statistically nonsignificant (Table 6).

In clinical stage II, the majority of the cases were using the quid for duration of 1–5 years and the cases showing OSF were more with the use of the processed quid. This association was found to be very highly significant (Table 7).

In clinical stage III, all the cases were using processed forms of areca nut and this association between type of quid and duration and frequency was found to be nonsignificant (Tables 8 and 9).

## 4. Discussion

The practice of chewing areca nut in a betel quid has a long history which is deeply ingrained in many sociocultural and religious activities, especially offered at the social and wedding gatherings. References to betel nut appear in ancient Sanskrit literature as early as the 1st century BC. The practice of chewing betel leaves after meals had become common (75 AD to 300 AD) and Sushruta, a renowned Indian physician, in his book* Mouth and Throat Diseases* mentioned a condition “Vidari,” that the features of which simulate OSF [3].

Oral submucous fibrosis is an insidious chronic disease affecting any part of the oral cavity or pharynx, occasionally proceeded by and/or associated with vesicle formation, and is always associated with a juxtaepithelial inflammatory reaction followed by progressive hyalinization of the lamina propria with epithelial atrophy leading to stiffness of the oral mucosa and causing trismus and inability to eat [6].

Areca nut, usually incorporated in betel quid, is the fourth most common psychoactive substance in the world after caffeine, alcohol, and nicotine and it has been estimated that betel quid is used by about 10–20% of the world's population accounting for up to 600 million areca nut users globally [7]. There have been numerous studies ascertaining the role of areca nut in oral cancer and stressing the importance and methods of screening and early detection of oral cancer thereby reducing the mortality rate [7–9]. A malignant transformation rate of 7.6% over a period of 17 years was previously reported; however, the recent studies indicate the malignant transformation rate to be 11.7% [8, 10].

The present study was conducted on 250 patients in and around Mangalore city and showed a male predominance which could be attributed to the social pattern of the study area wherein men get access to commercially available products more readily than females.

The 42.4% of cases in the present study were in age group of 26–35 years and this predominance among the younger generation could be attributed to wide social acceptability of areca nut chewing in the form of gutkha as compared to the traditional betel quid which is considered outdated. Further, the easy accessibility to the packaged products and the lack of knowledge about the carcinogenic effects of these products has led to increased use. Among the cases included in our study, 5.6% of the cases chewed type II quid, 7.2% of the cases chewed type III quid, 30.4% of the cases chewed processed areca nut, 39.2% of the cases chewed processed areca nut with processed tobacco, and 17.6% of the cases had multiple quid chewing habits (Figure 3). This was similar to the observations of Ahmad et al. [11] where around 55% chewed gutkha only and 16% of fibrosed cases were addicted to pan chewing habit. Goel et al. [12] in his study reported that 40% of patients chewed pan masala, 30% chewed betel nut, and the remaining 30% chewed betel quid.

This study showed an increased prevalence of the use of type V quid (processed areca nut and processed tobacco), attributed to the addictive effect associated with the usage of tobacco. Moreover, the habit is socially acceptable and these products are easily available which have led to its increased use.

## 5. Association between the Type of Quid and Clinical Staging of OSF

It was seen that, out of the 24 cases which showed clinical stage III grading, processed form of quid was used when compared to the cases where traditional forms of quid was used, which had clinical stage I and clinical stage II of OSF. This is attributed to the protective effects of the betel leaf against the harmful alkaloids present in the areca nut. The betel leaf is rich in beta-carotene and hydroxychavicol which have the capacity to quench the free radical that is toxic [11]. Another reason for the increased severity and risk of developing OSF in gutkha chewers is the increased dry weight of areca nut in gutkha sachets which is approximately 3.26 g, whereas that in betel quid is 1.14 g [13]. Therefore, the habitual gutkha users consume more dry weight of tobacco, areca nut, and slaked lime, which causes nicotine to act synergistically on the cytotoxicity induced by arecoline (a major areca nut alkaloid), thereby increasing the vulnerability of buccal mucosal fibroblasts to damage and enhancing collagen production up to 170%. There is also mechanical injury to oral tissues caused due to the fine grains of areca nut in the gutkha which allow ground tobacco to adhere to the traumatized mucosa, leading to morphologic changes and membrane damage. Thus, areca nut in combination with tobacco may cause cross-links and accelerate the onset of OSF in habitual gutkha chewers [14–16].

## 6. Association between the Type of Quid, Frequency, and Duration in Various Clinical Stages

In the present study, in clinical stage I, around 26 patients who chewed type IV quid for a frequency of 3–5/day developed OSF as compared to only 6 patients who consumed type II quid (Table 4).

Similarly, in clinical stage I, 26 subjects who chewed type IV quid for 1–5 years had OSF while those who used type II and type III quid with the betel leaf did not develop OSF within 1–5 years (Table 5).

In clinical stage II, around 18 patients who chewed type V quid for a frequency of 3–5/day had OSF as compared to only 2 who used type II quid (Table 6).

Similarly, 24 subjects who consumed type IV quid for 1–5 years had OSF while only 2 who consumed type II and type III quid for 1–5 years had OSF (Table 7).

In the above study, association between the clinical staging and duration was found to be highly significant as compared to the association between the clinical staging and frequency of quid usage. The above study also shows that the subjects who chewed type IV quid developed OSF earlier and those who chewed type IV quid with a greater frequency developed more severe clinical stages of OSF. This is similar to the observations done by Pandya et al. where maximum subjects who had histopathological grade III OSF took tobacco products for 8–10 years or with a frequency of 7–10 times per day [17]. Kumar et al. also suggested that the patients who used pan masala with a greater frequency/day developed OSF with a shorter duration of the habit [18].

However, the associations of various parameters in clinical stage III were not significant as the patients in clinical stage III were fewer in number (Tables 8 and 9).

## 7. Conclusion

Oral submucous fibrosis is a commonly occurring potentially malignant condition increasingly affecting the youth. The lack of knowledge of areca nut being a carcinogenic substance and the easy availability of gutkha and pan masala in social places have led to increased consumption and spread of this debilitating disease. The above study highlights the increased use of processed areca nut among the youth and varied styles of quid usage in terms of duration and frequency leading to severity of OSF in Mangalore population. There have been numerous studies and reviews on the various constituents of the quid and clinical aspects of OSF and oral cancer in India. This study serves as an extended methodology that can be used for screening and educating the masses about quid chewing habits and its association with oral cancer.

## Figures and Tables

**Table 1:** Haider et al. clinically graded OSF into the following.

Stage I	Faucial bands only
Stage II	Faucial and buccal bands
Stage III	Faucial, buccal, and labial bands

**Table 2 tab2:** Distribution of cases based on the type of quid.

Type of quid	Composition of the quid	Number of patients	Percentage
Traditional forms			
Type I	Betel leaf and areca nut	None	None
Type II	Betel leaf, areca nut and slacked lime	14	5.6%
Type III	Betel leaf, areca nut, lime, and tobacco	18	7.2%

Processed forms			
Type IV	Processed areca nut	76	30.4%
Type V	Processed areca nut and processed tobacco	98	39.2%

Type VI	Combined quid usage	44	17.6%

**Table 3 tab3:** Association between the type of quid and clinical staging.

Quid	Stage		
	I	II	III
Betel leaf and areca nut (0)	—	—	—
Betel leaf, areca nut, and slacked lime (14)	12 (85.71%)	2 (14.28%)	0
Betel leaf, areca nut, lime, and tobacco (18)	12 (66.66%)	6 (33.33%)	0
Processed areca nut (76)	44 (57.89%)	24 (31.57%)	8 (10.52%)
Processed areca nut and processed tobacco (98)	42 (42.85%)	42 (42.85%)	14 (14.28%)
Combined quid usage (44)	22 (50%)	20 (45.45%)	2 (4.54%)

*χ* ^2^ = 16.64; *p* = 0.034 SIG.

**Table 4 tab4:** Clinical stage I and frequency.

	1-2/day	3–5/day	6–10/day	>10/day
Type I	0	0	0	0
Type II	6 (4.5%)	6 (4.5%)	0	0
Type III	4 (3%)	4 (3%)	4 (3%)	0
Type IV	4 (3%)	26 (19.6%)	10 (7.5%)	4 (3%)
Type V	6 (4.5%)	16 (12.12%)	16 (12.12%)	2 (1.5%)
Combined	4 (3%)	16 (12.12%)	2 (1.5%)	0

*χ* ^2^ = 25.537; *p* = 0.0135 SIG.

**Table 5 tab5:** Clinical stage I and duration.

	1–12 months	1–5 years	5–10 years	>10 years
Type I	0	0	0	0
Type II	4 (3.0%)	0	2 (1.5%)	6 (4.5%)
Type III	4 (3.0%)	0	2 (1.5%)	6 (4.5%)
Type IV	14 (10.6%)	26 (19.6%)	2 (1.5%)	2 (1.5%)
Type V	10 (7.5%)	24 (18.18%)	8 (6.0%)	0
Combined	2 (1.5%)	12 (9.0%)	4 (3.0%)	4 (3.0%)

*χ* ^2^ = 53.504; *p* < 0.001 VHS.

**Table 6 tab6:** Clinical stage II and frequency.

	1 to 2/day	3 to 5/day	6–10/day	>10/day
Type I	0	0	0	0
Type II	0	0	2	0
Type III	0	2 (2.12%)	2 (2.12%)	2 (2.12%)
Type IV	2 (2.12%)	12 (12.76%)	8 (8.55%)	2 (2.12%)
Type V	2 (2.12%)	18 (19.14%)	12 (12.76%)	8 (8.55%)
Combined	0	8 (8.55%)	6 (6.3%)	6 (6.3%)

*χ* ^2^ = 12.89; *p* = 0.1675 NS.

**Table 7 tab7:** Clinical stage II and duration.

	1–12 months	1–5 years	5–10 years	>10 years
Type I	0	0	0	0
Type II	0	2 (2.12%)	0	0
Type III	0	2 (2.12%)	2 (2.12%)	2 (2.12%)
Type IV	2 (2.12%)	22 (23.4%)	0	0
Type V	8 (8.5%)	24 (25.53%)	8 (8.5%)	2 (2.12%)
Combined	0	8 (8.5%)	10 (10.6%)	2 (2.12%)

*χ* ^2^ = 30.692; *p* < 0.001 VHS.

**Table 8 tab8:** Clinical stage III and frequency.

	1 to 2/day	3 to 5/day	6–10/day	>10/day
Type I	0	0	0	0
Type II	0	0	0	0
Type III	0	0	0	0
Type IV	0	4 (16.6%)	2 (8.3%)	2 (8.3%)
Type V	0	4 (16.6%)	6 (25.0%)	4 (16.6%)
Combined	0	0	0	2 (8.3%)

*χ* ^2^ = 5.571; *p* = 0.2335 NS.

**Table 9:** Clinical stage III and duration.

	1–12 months	1–5 year	5–10 year	>10 years
Type I	0	0	0	0
Type II	0	0	0	0
Type III	0	0	0	0
Type IV	2 (8.3%)	2 (8.3%)	2 (8.3%)	2 (8.3%)
Type V	0	8 (33.33%)	4 (16.6%)	2 (8.3%)
Combined	0	2 (8.3%)	0	0

*χ* ^2^ = 1.979; *p* = 0.739 NS.
